# Tetanus or Hydrophobia.—Letter to Dr. E. S. Gaillard

**Published:** 1877-04

**Authors:** C. H. H. Fort

**Affiliations:** Adams Station, Tenn.


ADAMS STATION, TENN., Feb. 8, 1877.

*Dr. E. S. Gaillard*:

DEAR SIR,—I was called on the morning of the 6th of August last, at 5 o'clock A. M., to see a negro boy, five miles distant, aged nine years. The father came for me, and informed me that his son had jumped down from a high rail fence upon a lot of loose boards and stuck a nail in his heel, but pulled it out without breakit off in his foot. The accident happened on Friday, and I saw him on Monday morning. His mother informed me that at supper time the night before he complained of feeling badly. He eat his supper, but soon threw it us, and complained of his back, head, and stomach, and also said his heel hurt him. As is usual with the negro, the parents were "sleepy-headed," and went to sleep, and about midnight he commenced making a very strange noise. After lighting a lamp, the boy called for a drink of water, and when put to his mouth it threw him into a severe paroxsm. They became much alarmed. The paroxysm did not last a great while, and the patient begged his mother again for water, with the same effect. From ths information gained from his mother, I suppose he must have had a dozen paroxysms before I arrived.

Upon examination, I found his heel had not much swollen, presenting the appearance of what is commonly called a stone-bruise. It had been well poulticed. I made a free crucial incision. It did not seem to give him much pain. He was entirely rational during the intervals intervening the paroxysms. There was but little pus in his heel. His heel had been "picked up" so by his mother that I could not discover the track of the nail. His contortions and gyrations being so peculiar that my doubts arose as to it being a case of tetanus, and Ibegan to experiment to see if I could get up a case of hydrophobia. I found that the slightest puff of my breath would produce a hideous paroxysm. He presented the appearance of trying to "grin out of his hide." I tried him water with the same effect. I cauterized his split heel freely with carbolic acid. He did not complain. I then endeavored to chloroform my patient, but as soon as I would place the napkin over his face he would bounce and twist so that I could not, with assistanee, hold it over his face, and after several trials had to give up without ever getting my patient under the influence of chloroform. The pouring of water into a basin would produce paroxysm. I never succeeded in getting a particle of medicine down him, and I gave him up as a "lost negro." He died sometime during the night, and it was rather strange to think that such an immense quantity of saliva could be emitted in such a short time. It was tough and viscid. Opisthotonos was also plainly developed, and risus sardonicus also, which symptoms belong to tetanus.

In giving the history of this case, I am prompted by a two-fold desire. In the first place, I claim to be now and have been a student of medicine for several years, and consequently I want a little edification upon the subject. Does traumatic tetanus ever produce such a train of symptoms as described above? I confess I have never read of such. I have been forced to conclude that tetanus brought to light, as it were, the latent germ of hydrophobia that was dormant in his system, and that my patient perhaps had both diseases. The patient was entirely rational when not in a paroxysm, and would beg for water and talk very rationally for a negro of his age. There were intermissions between the paroxysms, sometimes of thirty minutes, I should suppose. I never saw a human being afflicted with or die of hydrophobia. I have seen dogs, hogs, cows, and one horse die of hydrophobia. I confess my inability to draw a line of demarcation between traumatic tetanus and hydrophobia so as to know, after studying the history of both closely, which killed my patient. He had never been bitten by a dog, as far as I know. Respectfully,

C. H. H. Fort, M. D.

—*Am. Med. Bi-Weekly.*

